# Transcriptome and Deletion Mutant Analyses Revealed that an RpoH Family Sigma Factor Is Essential for Photosystem Production in *Roseateles depolymerans* under Carbon Starvation

**DOI:** 10.1264/jsme2.ME22072

**Published:** 2023-03-07

**Authors:** Tetsushi Suyama, Nanako Kanno, Satoko Matsukura, Kotaro Chihara, Naohiro Noda, Satoshi Hanada

**Affiliations:** 1 Bio-Analytical Research Group, Biomedical Research Institute, National Institute of Advanced Industrial Science and Technology (AIST), Tsukuba, Ibaraki, Japan; 2 Photosynthetic Microbial Consortia Laboratory, Department of Biological Sciences, Graduate School of Science, Tokyo Metropolitan University, Hachioji, Tokyo, Japan; 3 Department of Life Science and Medical Bioscience, Waseda University, Shinjuku, Tokyo, Japan

**Keywords:** purple bacteria, aerobic anoxygenic phototroph, starvation, transcriptome, photosynthetic gene cluster, sigma factor, RpoH

## Abstract

*Roseateles depolymerans* is an obligately aerobic bacterium that produces a photosynthetic apparatus only under the scarcity of carbon substrates. We herein examined changes in the transcriptomes of *R. depolymerans* cells to clarify the expression of photosynthesis genes and their upstream regulatory factors under carbon starvation. Transcriptomes 0, 1, and 6‍ ‍h after the depletion of a carbon substrate indicated that transcripts showing the greatest variations (a 500-fold increase [6 h/0 h]) were light-harvesting proteins (PufA and PufB). Moreover, loci with more than 50-fold increases (6 h/0‍ ‍h) were fully related to the photosynthetic gene cluster. Among 13 sigma factor genes, the transcripts of a sigma 70 family sigma factor related to RpoH (SP70) increased along photosynthesis genes under starvation; therefore, a knockout experiment of SP70 was performed. ΔSP70 mutants were found to lack photosynthetic pigments (carotenoids and bacteriochlo­rophyll *a*) regardless of carbon starvation. We also examined the effects of heat stress on ΔSP70 mutants, and found that SP70 was also related to heat stress tolerance, similar to other RpoH sigma factors (while heat stress did not trigger photosystem production). The deficient accumulation of photosynthetic pigments and the heat stress tolerance of ΔSP70 mutants were both complemented by the introduction of an intact SP70 gene. Furthermore, the transcription of photosynthetic gene operons (*puf*, *puh*, and *bch*) was markedly reduced in the ΔSP70 mutant. The RpoH homologue SP70 was concluded to be a sigma factor that is essential for the transcription of photosynthetic gene operons in *R. depolymerans*.

When nutrient limitations occur, microbes change their mode of metabolism to more favorable states for survival. Some microbes slow down their respiration and growth rates or go into dormancy (Bergkessel *et al.*, 2016; Gray *et al.*, 2019), some begin to utilize alternative substrates for life maintenance (Görke and Stülke, 2008; Bergkessel *et al.*, 2016), some increase virulence to utilize other organisms as a source of nutrients (Görke and Stülke, 2008), and some take advantage of mutagenesis for long-term survival (Finkel and Kolter, 1999; Finkel, 2006; Bergkessel *et al.*, 2016; Chib *et al.*, 2017). A subunit of prokaryotic RNA polymerase called a sigma factor, which recognizes gene promotor sequences on the genome, enables the initiation of specific gene transcription by generating a holoenzyme complex. Several sigma factor isomers modulate the transcriptional sets of genes and are called regulons. In *Escherichia coli*, RpoD (or σ^70^) is the main sigma factor in exponentially growing cells, while alternative sigma factors activate stress-adaptive regulons. RpoS (or σ^38^) is one of the alternative sigma factors responsible for the expression of stress-response genes in addition to some metabolic genes in the stationary phase and under starved conditions, and may directly or indirectly contribute to the adaptive responses to starvation described above (Bergkessel *et al.*, 2016; Wong *et al.*, 2017). RpoH (or σ^32^) is another example of an alternative sigma factor that is responsible for the transcription of genes for the heat-shock response (Feklístov *et al.*, 2014), while additional functions, such as overcoming photooxidative stress, have also been reported for some species (Du and Arvidson, 2006; Barnett *et al.*, 2012; Dufour *et al.*, 2012; Kumar *et al.*, 2012; da Silva Neto *et al.*, 2013; López-Leal *et al.*, 2016; Sasaki *et al.*, 2016; Mitsui and Minamisawa, 2017).

*Roseateles depolymerans* (Suyama *et al.*, 1999) is a betaproteobacterium with similar characteristics to those of aerobic anoxygenic phototrophic (AAP) bacteria belonging to *Alphaproteobacteria* (Shimada, 1995; Hanada, 2016). AAP bacteria are strictly aerobic heterotrophs that only grow by respiration; however, they also produce a photosynthetic apparatus. The reason why they produce a photosynthetic apparatus was originally controversial. However, the phototrophy of AAP bacteria has been demonstrated in many species (Yurkov and van Gemerden, 1993; Koblížek *et al.*, 2010; Kirchman and Hanson, 2013; Bill *et al.*, 2017). *R. depolymerans* produces a photosynthetic apparatus *de novo* only under the shortage of carbon substrates for respiration (*e.g.*, sugars, amino acids, and organic acids), whereas its growth is observed non-phototrophically only under the presence of carbon substrates without the production of a photosynthetic apparatus (Suyama *et al.*, 2002). The production of a photosynthetic apparatus appears to require a sudden decrease in a carbon substrate from rich to poor because entering into the stationary phase via the slow consumption of carbon substrates (or an artificially produced moderate decrease in the concentration of carbon) did not trigger this phenomenon (Suyama *et al.*, 2002). The up-regulation of photosynthesis in the presence of a low concentration (or its down-regulation with a high concentration) of carbon substrates is not limited to *R. depolymerans*. Kolber *et al.* (2001) reported an increase in the photosynthetic electron transport of oceanic isolates in organic-poor medium and a decrease in organic-rich medium. Hirose *et al.* also demonstrated that isolates of freshwater AAP bacteria in organic-poor medium showed the higher production of bacteriochlo­rophyll (BChl) *a* (Hirose *et al.*, 2016). Increases in longevity with light illumination under carbon starvation were observed in *R. depolymerans*, and we previously concluded that *R. depolymerans* utilized light energy for survival only during a scarcity of respiratory substrates (Suyama *et al.*, 2002). The utilization of light energy for survival under long-term starvation has also been demonstrated for the AAP bacterium *Dinoroseobacter shibae* (Soora and Cypionka, 2013; Soora *et al.*, 2015). The use of a photosynthetic apparatus for long-term survival under carbon starvation has also been suggested for some phototrophic (but not AAP) bacteria (Breznak *et al.*, 1978; Oda *et al.*, 2000; Kanno *et al.*, 2014; Kanno *et al.*, 2018). The photosynthetic apparatus in these AAP and non-AAP bacteria is considered to act as an auxiliary supplier of electron transport under the starvation of respiratory substrates.

The photosynthesis genes in purple bacteria (including AAP bacteria) comprise *puf*, *puc*, and *puh* operons coding for the apoproteins of a photosynthetic reaction center complex and light harvesting complexes, a *bch* operon encoding the enzymes for the biosynthesis of BChls, and a *crt* operon encoding the enzymes for the biosynthesis of carotenoids. Most of the photosynthetic operons are arranged in a large ‘photosynthetic gene cluster (PGC).’ The structures of PGCs are conserved, and the horizontal transfer of PGCs in the history of evolution has been suggested (Igarashi *et al.*, 2001; Zheng *et al.*, 2011; Nagashima and Nagashima, 2013; Brinkmann *et al.*, 2018).

The transcriptional regulatory systems of photosynthesis genes have been extensively examined in *Rhodobacter capsulatus* and *Rhodobacter sphaeroides* (Buggy *et al.*, 1994; Bauer *et al.*, 2003; Masuda and Bauer, 2004). Two-component regulatory systems, RegB-RegA (*R. capsulatus*) and PrrB-PrrA (*R. sphaeroides*), up-regulate photosynthesis genes in the absence of oxygen (Bauer *et al.*, 2003). The DNA-binding proteins, CrtJ (*R. capsulatus*) and PpsR (*R. sphaeroides*), have been shown to repress photosynthesis genes under aerobic conditions (Bauer *et al.*, 2003). PpsR also acts as a high light repressor, whereas CrtJ does not (Bauer *et al.*, 2003). AppA (*R. sphaeroides*) stimulates photosynthesis genes by inhibiting PpsR in the absence of oxygen as well as blue light by two distinct mechanisms (Bauer *et al.*, 2003). AerR (*R. capsulatus*), an antirepressor for CrtJ (coded by an immediately upstream ORF of *crtJ*) has two translational variants (long and short) that differentially bind to vitamin B_12_ in the presence and absence of light, and up-regulate *bch*, *crt*, and *puc* genes in the light (Fang and Bauer, 2017; Yamamoto *et al.*, 2018). PpaA (an AerR homolog for *R. sphaeroides*) also acts as an antirepressor for PpsR, but has no variants for sensing light via vitamin B_12_ binding, and its regulation is inconspicuous behind the regulation by AppA (Vermeulen and Bauer, 2015; Yamamoto *et al.*, 2018). HvrA (*R. capsulatus*) activates *puf* and *puh* operons under dim light (Buggy *et al.*, 1994) and may also relate to a stringent response because a gene that is essential for the formation of ppGpp was only mutated against the background of Δ*hvrA* (Masuda and Bauer, 2004). The RNA chaperone Hfq has been suggested to stabilize the mRNAs of the *puf* and *puc* genes in *R. sphaeroides* (Berghoff *et al.*, 2011b). However, the regulatory systems responding to carbon starvation have not yet been reported for photosynthesis genes.

The complete genome sequence of *R. depolymerans* strain KCTC 42856^T^ (=61A^T^) recently reported by Lee *et al.* (2016) indicates the presence of PGC, as shown in Fig. 1. The structure of PGC was similar to those reported for other species (Igarashi *et al.*, 2001; Zheng *et al.*, 2011; Nagashima and Nagashima, 2013; Brinkmann *et al.*, 2018); however, light harvesting complex II genes (*puc*) were not found in the *R. depolymerans* genome (Lee *et al.*, 2016). Among previously identified regulators for PGC, a *ppaA* gene, *ppsR* gene, and *crtJ* homologue (ORF350 [Suyama *et al.*, 2002]) were detected in the *R. depolymerans* PGC; however, their contribution to the regulation of PGC remains unknown. The accumulation of BChl *a* and the mRNAs of *pufBA* genes were previously observed in *R. depolymerans* cells under artificially produced carbon starvation conditions that were maintained for more than 27 and 72 h, respectively, but not in non-starved cells (Suyama *et al.*, 2002). The earlier responses of *pufBA* and other genes currently remain unclear. Therefore, the present study examined regulatory pre-phenotypic changes from the respirational to phototrophic phases via a whole transcriptome ana­lysis, with a focus on early responses after a decline in the concentration of carbon, and investigated the mechanisms underlying the sensing of carbon starvation and subsequent metabolic changes in *R. depolymerans*. We identified a few sigma factor genes (SP70 and SPECF) transcribed under carbon starvation that may be associated with photosynthesis gene regulation; accordingly, knockout experiments of these genes were performed. We herein report the discovery of a sigma factor that is expressed under carbon-starved conditions and is essential for the production of a photosystem in *R. depolymerans*.

## Materials and Methods

### Bacterial strain and culture conditions

We used succinate as a carbon source in the present study (instead of casamino acids used in previous studies [Suyama *et al.*, 1999; Suyama *et al.*, 2002]) because casamino acids are a mixture of chemicals and their complex metabolism makes them unsuitable for discussing simple carbon starvation. Succinate is a promising carbon source for inducing *R. depolymerans* to elicit the same starvation responses as previously reported (Suyama *et al.*, 2002). The composition of C-free medium was the same as that previously described (Suyama *et al.*, 2002). 0.4SAV (0.4% [w/v], succinate with ammonium and vitamins) and 0.02SAV (0.02% [w/v], succinate with ammonium and vitamins) media were prepared by adding 4‍ ‍g‍ ‍L^–1^ and 0.2‍ ‍g‍ ‍L^–1^ sodium succinate, respectively, to C-free medium. To construct the mutant strains, a 0.2CAV (0.2% [w/v], casamino acids with ammonium and vitamins) medium (Suyama *et al.*, 1999) was also used. The strains and plasmids used in the present study are listed in Table 1. All strains were preserved in a –80°C freezer in the form of a glycerol stock (0.5‍ ‍mL of the culture at the exponentially growing phase was mixed with 0.5‍ ‍mL 50% [w/v] glycerol, and frozen in liquid nitrogen), thawed on ice before the initiation of each experiment, and grown in 0.4SAV. Mutant strains were maintained with appropriate antibiotics (ampicillin sodium salt, 100‍ ‍mg L^–1^ and kanamycin sulfate salt, 50‍ ‍mg L^–1^). Ten milliliters of the culture was placed in an L-shaped tube (Φ18×120×70H mm) and incubated at 30°C in the dark with shaking at 45 strokes min^–1^ in a Personal-11SD water bath shaker equipped with an MD-1218 Monod-type shaking platform (TAITEC). Cultures were also performed at 37, 40, and 42°C in the same manner as described above.

### Carbon starvation conditions

The cells of strain 61A^T^ (or strain 1AA1) grown at 30°C for 20–24‍ ‍h in 0.4SAV (absorbance reading at 660‍ ‍nm (*A*_660_)≈0.35, the exponentially growing phase) were harvested by centrifugation (2,940×*g*, 5‍ ‍min), washed twice with 50‍ ‍mL of ice-cold C-free medium, and then suspended into 0.02SAV. The cell suspension was adjusted to *A*_660_ of 0.35. Ten-milliliter aliquots of the cell suspension were placed in L-shaped tubes and incubated again at 30°C for the indicated periods. Cells before being resuspended in 0.02SAV were collected for the non-starved sample (0‍ ‍h). Cells after 1 and 6‍ ‍h of incubation with 0.02SAV (growth was arrested as previously reported [Suyama *et al.*, 2002]) were also harvested. All experimental operations for changing the medium and collecting cells were conducted with chilling on ice. The accumulation of carotenoids and BChl *a* is not a cold-induced effect because cells returned to fresh 0.4SAV after the chilling operation did not exhibit this phenomenon (Suyama *et al.*, 2002).

### Spectroscopic ana­lysis

Pigments were extracted as previously reported (Suyama *et al.*, 2002). Absorption spectra were recorded with a GeneQuant 1300 spectrophotometer (GE Healthcare Bio-Sciences).

### Transcriptome ana­lysis

Total RNA was extracted using an ISOGEN kit (based on the liquid phase-separation method after lysis with a reagent containing phenol and guanidine salt; NIPPON GENE). Total RNAs extracted from 20-mL cultures (two tubes of 10-mL cultures) for each condition were 97.5–125.7‍ ‍μg with RIN (RNA integrity number; Schroeder *et al.*, 2006) of 7.7–8.0, and were successfully sequenced. No replication was sequenced because this experiment was conducted simply as a survey of the candidate loci. The following 4 steps were performed by Hokkaido System Science: 1. Total RNA was treated with the Ribo-Zero rRNA Removal Kit for Bacteria (Illumina) and the library was prepared using the TruSeq Stranded mRNA Sample Prep Kit (Illumina) with the standard protocol. 2. Size selection (>200 bp) was performed using the Agencourt AMPure XP Kit (Beckman Coulter Genomics). 3. Sequencing was conducted on the HiSeq sequencing system (Illumina) with paired-end runs. 4. Ten million raw sequencing reads were recorded for each sample (=five million read pairs, adapter sequences removed, 100 nucleotides per read). Sequences were analyzed using CLC Genomics Workbench 11.0.1 (www.qiagenbioinformatics.com). Raw sequencing reads were quality checked. Low-quality sequences (limit of error probability <0.01, maximal number of ambiguous nucleotides allowed in the sequence after trimming=0) and reads shorter than 50 were trimmed for subsequent ana­lyses. The remaining reads were mapped to‍ ‍coding sequences (CDS) in the genome of *R. depolymerans* strain KCTC 42856^T^ (=61A^T^) (GenBank accession number NZ_CP013729: Ver. NZ_CP013729.1). (Optional: the Ribo-Zero rRNA Removal Kit [Bacteria] is no longer available for the RNA sequencing of 6-h starved cells of mutant ΔSP70 strain 1AA1; therefore, the RiboMinus Transcriptome Isolation Kit, Bacteria [Thermo Fisher Scientific] was used at Hokkaido System Science. Regarding comparisons with other transcriptome data, we initially mapped the reads to CDS for rRNA operons [RD2015_RS08385 to RD2015_RS08420, nucleotide region 1971529–1982537; and RD2015_RS22130 to RD2015_RS22165, nucleotide region 5167698–5178704] and then mapped the unmapped reads to CDS in the *R. depolymerans* genome.) Gene counts and Transcripts Per Million (TPMs) (Wagner *et al.*, 2012) for each gene were obtained. To construct heatmaps with EPS images, further ana­lyses were conducted using R software (version 3.6.3) run on RStudio (1.2.5042). Gene counts were normalized by the Trimmed Mean of M-values (TMM) method using Bioconductor R package edgeR (version 3.28.1), and TMM-normalized log counts per million (log2 CPM) were calculated. Normalized log2 CPM values were converted to Z-scores using the R package genefilter (version 1.68.0) and a heatmap of Z-score data was created with the heatmap.2 function from R package gplots (3.1.1). Transcriptome data for all loci used to construct Fig. 2 and 3 are summarized in Table S1.

### Construction of ΔSP70 and ΔSPECF mutants

The region containing SP70 and SPECF (including non-coding regions flanked by start and stop codons for up- and downstream ORFs) were PCR amplified from a genomic DNA extract from *R. depolymerans* 61A^T^ (Suyama *et al.*, 1999) using Pyrobest DNA Polymerase (Takara Bio). The specific primer pairs fused to the *Xba*I and *Kpn*I sites, namely, 5′-tctctagatgaacactgtgtgcaagcta and 5′-taggtaccatggagtctccagactccga for SP70 and 5′-tctctagaaggcgggttgcggtggagcg and 5′-taggtaccatggttcggtggatgtcact for SPECF, were used for amplification. Amplicons were cloned into the *Kpn*I–*Xba*I site of the pUC19 vector (pUC19SP70_1-2 and pUC19SPECF_2-7 in Table 1). Clones were digested with *Msc*I and *Ngo*MIV (SP70) or *Eco*RV and *Bsp*EI (SPECF). A 1.3-kb fragment containing the kanamycin resistance (Km^r^) gene was excised from the pSUP5011 plasmid (Suzuki *et al.*, 2002) using *Hin*dIII (blunt ended using Pyrobest polymerase) and *Xma*I, and ligated into the 3.7-kb (SP70) or 3.1-kb (SPECF) digests of the plasmids (pUC19ΔSP70K_1-1 and pUC19ΔSPECFK_2-6 in Table 1). The *Eco*RI–*Xba*I fragments containing the SP70 and SPECF genes disrupted by the insertion of the Km^r^ gene were cloned into the pG19II suicide vector (pG19IIΔSP70K_1-A and pG19IIΔSPECFK_2-C in Table 1). The ‘disruptor’ constructs were electroporated into *R. depolymerans* 61A^T^. Single crossover mutants were screened using 0.2CAV agar plates containing kanamycin and 10‍ ‍mg L^–1^ gentamicin sulfate salt. The ΔSP70 and ΔSPECF clones (double crossover mutants) were screened using 0.2CAV agar plates containing kanamycin and 5% (w/v) sucrose.

### Complementation of ΔSP70 mutants

The pUC19SP70_1-2 plasmid (Table 1) was electroporated into the ΔSP70 mutants (1AA1 and 1AB1) and screened using agar plates containing ampicillin and kanamycin. Mutants harboring the pUC19SP70_1-2 plasmid on the genome (A4h and B7h) were maintained in the presence of antibiotics.

### RNAseq data accession numbers

Transcriptome sequencing data have been deposited in the DDBJ/EMBL/GenBank data libraries with the accession numbers SAMD00409672, SAMD00409673, SAMD00409674, and SAMD00409675.

## Results

### Changes in the transcriptome under carbon starvation conditions

**Fig. 1.**
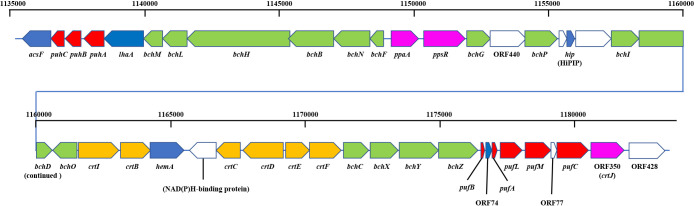
Arrangement of a photosynthetic gene cluster predicted from the nucleotide sequence of the *Roseateles depolymerans* genome reported by Lee *et al.* (2016). The lateral scale represents nucleotide positions referenced to genome data. Genes are presented as arrows pointing in the direction of their transcription. Red, *puf* and *puh* genes; green, *bch* genes; orange, *crt* genes; pink, regulator genes; blue, genes conserved with the photosynthetic gene clusters of other species; and blank, uncertain, or unrelated genes.

**Fig. 2.**
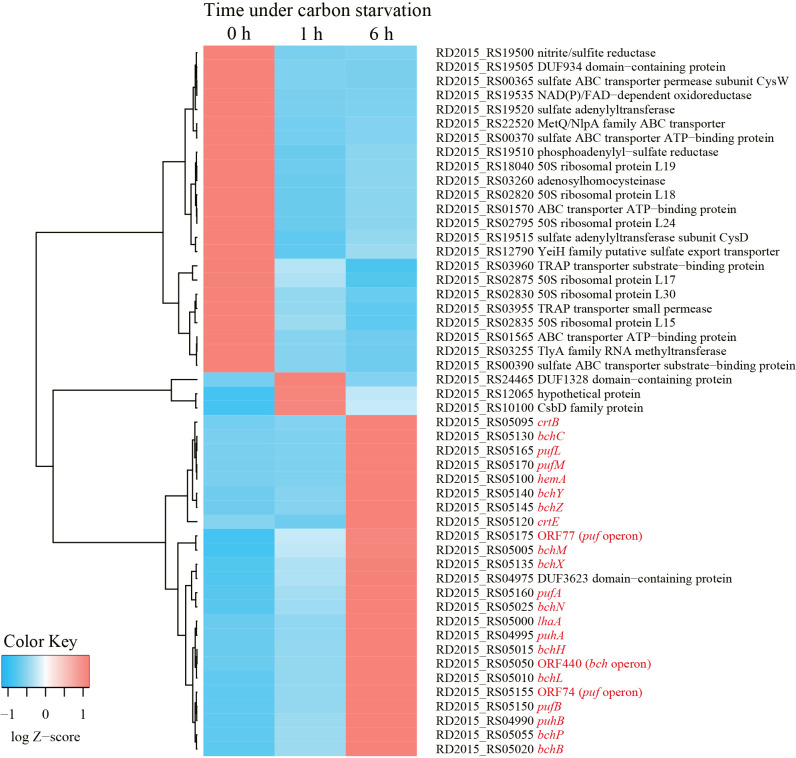
A heat map showing 50 genes exhibiting the greatest transcript variations in *Roseateles depolymerans* 61A^T^ cells after 0, 1, and 6‍ ‍h of carbon starvation. Genes with low abundance (lower than 10 counts in all three samples) were omitted. The profiles of 0-, 1-, and 6-h starved cells are shown in that order in the leftmost columns. Genes exhibiting similar changes in abundance are grouped on the left. The locus tag, locus name, and annotations of genes are indicated on the right. Genes related to the photosynthetic gene cluster are indicated with red letters.

**Fig. 3.**
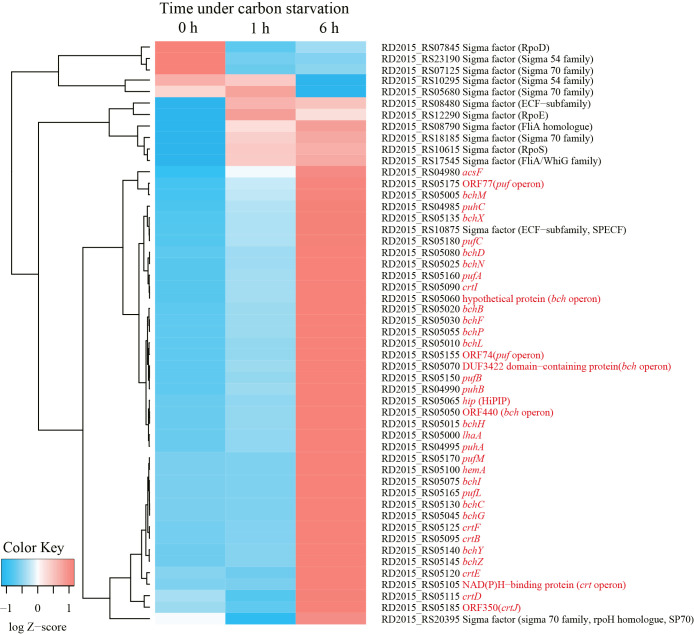
A heat map showing 13 sigma factor genes and genes related to the photosynthetic gene cluster in transcripts of *Roseateles depolymerans* 61A^T^ cells after 0, 1 and 6‍ ‍h of carbon starvation. The profiles of 0-, 1-, and 6-h starved cells are shown in that order in the leftmost columns. Genes exhibiting similar changes in abundance are grouped on the left. The locus tag, locus name, and annotations of genes are indicated on the right. Genes related to the photosynthetic gene cluster are indicated with red letters.

Phenotypically, the accumulation of carotenoids and BChls was not observed in wild-type *R. depolymerans* (strain 61A^T^) cells grown in succinate-base organic-rich medium (0.4SAV) and was not clear in those starved in organic-poor medium (0.02SAV) for less than 27‍ ‍h (but became obvious in those starved for more than 45‍ ‍h). To observe earlier responses in transcriptional levels, we focused on cells starved for 1 and 6‍ ‍h and compared them with non-starved cells. Fig. 2 shows the 50 loci with the greatest variations in RNA sequence abundance for the samples at 0, 1, and 6 h. The transcripts abundant in exponentially growing cells in 0.4SAV (red tiles in the upper left column) were characterized by genes for ribosomal proteins, components relating to transporters across membranes, and enzymes for sulfur and energy metabolism, whereas the majority of transcripts abundant in 6-h starved cells (red tiles in the lower right column) were the genes related to PGC (indicated with red letters in Fig. 2, cf. Fig. 1). There are 13 sigma factor genes in the *R. depolymerans* genome, and they may differentially control the expression of genes under starvation conditions. Fig. 3 shows a heat map of the 13 sigma factor genes with the genes related to PGC (indicated with red letters in Fig. 3). All photosynthesis gene transcripts were shown to be abundant in 6-h starved cells, but not in 0- or 1-h starved cells. At the top of the heat map, five sigma factor transcripts were found to be abundant in exponentially growing cells (red tiles in the upper left column). Three sigma factor transcripts including ‘RpoD’ disappeared in 1-h starved cells, and the remaining two sigma factor transcripts abundant in 0- to 1-h starved cells also disappeared in 6-h starved cells. Six additional sigma factor transcripts including those for RpoE and RpoS appeared after 1‍ ‍h of starvation, and remained abundant in 6-h starved cells. Two more sigma factor transcripts appeared in 6-h starved cells: one was for an ECF-subfamily sigma factor (RD2015_RS10875) clustered with photosynthesis genes, while the other was for a sigma 70 family sigma factor related to RpoH (RD2015_RS20395) clustered near the root of photosynthesis gene transcripts. We provisionally designated the ECF-subfamily sigma factor as ‘SPECF’ (named after ‘Succinate Privation’ and ‘ECF-subfamily’) and the sigma 70 family sigma factor as ‘SP70’ (named after ‘Succinate Privation’ and ‘sigma 70 family’) and examined the deletion and complementation of the two sigma factor genes.

### SP70 and SPECF mutants

**Fig. 4.**
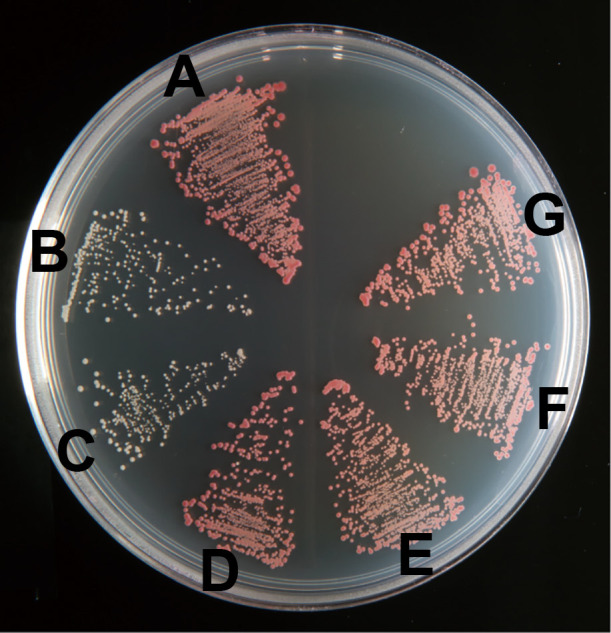
Colonies of *Roseateles depolymerans* wild-type strain 61A^T^ (A); ΔSP70 mutants, 1AA1 (B) and 1AB1 (C); ΔSPECF mutants, 2CA3 (D) and 2CA4 (E); and SP70-complemented mutants on the ΔSP70 background, A4h (F) and B7h (G). Strains were precultured in 0.4SAV liquid medium with or without antibiotics and then streaked onto the non-selective 0.02SAV agar plate. The photograph was taken 14 days after an incubation at 30°C in the dark.

**Fig. 5.**
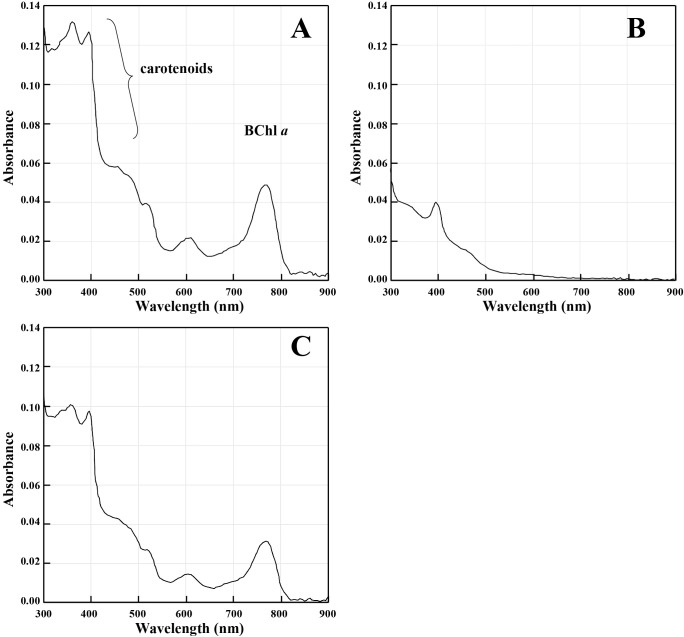
Absorption spectra of methanol extracts from cells of the wild-type strain, 61A^T^ (A); a ΔSP70 mutant, 1AA1 (B) and an SP70-complemented mutant, A4h (C) incubated under carbon-starved conditions for 50‍ ‍h in the dark. The intensities of spectra were normalized for the dry weight of the sample cells. The assignment of absorption peaks for bacteriochlo­rophyll *a* and carotenoids is shown in panel (A). All cells were precultured up to *A*_660_ of 0.35 in 0.4SAV with or without antibiotics and then resuspended (*A*_660_ of 0.35) and incubated in non-selective 0.02SAV.

The wild-type (61A^T^), ΔSP70 (1AA1 and 1AB1), ΔSPECF (2CA3 and 2CA4), and SP70-complemented (A4h and B7h) strains that colonized on the 0.02SAV agar plate are shown in Fig. 4. The colonies of ΔSP70 strains (Fig. 4B and C) appeared to be white, while those of the wild-type (Fig. 4A), ΔSPECF (Fig. 4D and E), and SP70-complemented (Fig. 4F and G) strains were pink (Fig. 4). Colonies for all strains were visible 1 day after streaking on agar, and colonies for the wild-type, ΔSPECF, and SP70-complemented strains were faintly pink 2 days thereafter. The ΔSP70 strains did not exhibit a pink color throughout one month of incubation. Phenotypic changes were not observed in the ΔSPECF strains and we concluded that this sigma factor was not related to the transcription of photosynthesis genes under carbon starvation. The absorption spectra of methanol extracts from the wild-type (61A^T^), ΔSP70 (1AA1), and SP70-complemented (A4h) strains are shown in Fig. 5A, B, and C. The accumulation of BChl *a* and carotenoid pigments was eliminated in the ΔSP70 mutant (Fig. 5B) and was complemented with the SP70 gene (Fig. 5C).

### Transcriptome ana­lysis of the ΔSP70 mutant

All 24 genes shown in Fig. 2 that were up-regulated in cells starved for 6‍ ‍h were reduced, while the other 26 genes were unchanged or slightly increased in the ΔSP70 mutant. Table 2 lists the genes with expression levels that were markedly lower in the ΔSP70 mutant (1AA1) under 6‍ ‍h of starvation than in the wild-type strain (61A^T^). The transcription of the remaining regions of the SP70 gene (386 nts upstream of the *Msc*I site and 48 nts downstream of the *Ngo*MIV site) was detected in the ΔSP70 mutant (marked with ‘*’, 17.81% of the count in the wild-type strain). Approximately 50% of the affected genes were related to PGC and were identified as the genes responding to carbon starvation listed in Fig. 2 and 3. The top three loci, *pufA*, *pufB*, and ORF74, were co-transcribed as a major 1-kb transcript of the *puf* operon (Suyama *et al.*, 2002) and showed the greatest changes in the wild-type strain under carbon starvation conditions (Fig. 2). Besides the *puf*, *puh*, and *bch* genes, a *hip* gene (RD2015_RS5065) coding for high-potential iron-sulfur protein (HiPIP) may also be an essential component of the *Roseateles* PGC because HiPIP has been suggested as a major electron donor to the photosynthetic reaction center in *Rubrivivax gelatinosus*, a phototrophic bacterium phylogenetically related to *R. depolymerans* (Nagashima *et al.*, 2002). Some loci related to type II toxins, which may affect stringent responses, also declined with the depletion of SP70. Most of the other genes were functionally unknown (Table 2). The impact of the SP70 deletion on *ppaA*, *ppsR*, all *crt* genes, and some *bch* genes (positioned between *hip* and *crt* genes, cf. Fig. 1) was minimal. Increases in transcripts due to the deletion of SP70 were also implied for the *crtD* and *crtC* genes and the gene for the NAD(P)H-binding protein coded in PGC.

### Heat tolerance of SP70 mutants

Since RpoH is related to heat shock responses in other organisms, differences in heat tolerance between wild-type and SP70 mutants were also examined. The growth abilities of the wild-type (61A^T^), ΔSP70 (1AA1 and 1AB1), and SP70-complemented (A4h and B7h) strains in 0.4SAV at 30, 37, 40, and 42°C are summarized in Table 3. All strains showed almost the same growth curves with similar doubling times and maximum growth levels at 30°C. The wild-type and SP70-complemented strains also grew with similar growth rates at 37, 40, and 42°C, while they appeared to be impaired at 42°C. The ΔSP70 strains did not grow at 40 or 42°C. SP70 was found to be related to heat tolerance, as has been reported for RpoHs in other organisms. Although the wild-type strain grew under SP70 expression in 0.4SAV medium at 42°C, no BChl *a* or carotenoid pigments were detected, suggesting that photosynthetic genes were not expressed under this condition (Fig. S1).

## Discussion

The up-regulation of *de novo* synthesis under the lack of energy sources is not advantageous unless the product is helpful for survival. As we reported previously, *R. depolymerans* produces a photosystem *de novo* only after a decrease in carbon sources, mainly with the depletion of amino acids as substrates (Suyama *et al.*, 2002). This phenomenon was confirmed in the present study using succinate as a simpler carbon source, and the results obtained also showed that the majority of genes up-regulated by more than 50-fold under carbon starvation were related to the production of a photosystem (Fig. 2). The abundance of *pufB* and *pufA* gene transcripts increased 500-fold under these conditions. The mechanisms underlying the super transcription of this operon warrants further study. Yin *et al.* (2019) reported that another anoxygenic phototroph, *Rhodopseudomonas palustris*, maintained protein synthesis in a growth-arrested state and utilized photosynthetic energy conversion for the longevity of cells. The difference between the two species is that the photosynthesis of *R. depolymerans* appears to be specialized for survival, while that of *R. palustris* supports growth.

The ΔSP70 strains were deficient at accumulating photosynthetic pigments (Fig. 4B, C, and 5B). The transcription of the loci listed in Table 2 was markedly depressed, suggesting that the *puf*, *puh*, and some *bch* genes were directly or indirectly controlled by SP70 as a regulon. Changes in the expression of these genes are considered to directly affect the phenotype. Therefore, SP70 is considered to be an essential sigma factor for photosystem production in *R. depolymerans* under carbon starvation. Although the transcription of the majority of genes in PGC was stimulated under carbon starvation (Fig. 2 and 3) and depressed by the depletion of the SP70 gene (Table 2), the *crt* and *bch* genes as well as the *ppaA* and *ppsR* genes (which are considered to act as the repressor and antirepressor, respectively, for the *crt* and *bch* genes) positioned between *bchI* and *crtF* (cf. Fig. 1) were not depressed in the ΔSP70 strain (Table 2). There appear to be other transcriptional system(s) for the *crt*, *bch*, *ppaA*, and *ppsR* genes that work together with the SP70 regulon, but transcribe them in the absence of the SP70 sigma factor.

RpoH, a known homologue of SP70, is an alternative sigma factor for the ‘heat shock’ response in many bacterial species (Dufour *et al.*, 2012; López-Leal *et al.*, 2016). Although some promoter sequences recognized by RpoH are conserved in other species (Dufour *et al.*, 2012; López-Leal *et al.*, 2016), we did not identify similar sequences in *R. depolymerans* PGC. In the near future, we intend to examine the promoter recognition of SP70. Some RpoHs have been shown to have additional regulatory functions, *e.g.*, the up-regulation of genes involved in iron uptake (da Silva Neto *et al.*, 2013), the establishment of symbiosis (Sasaki *et al.*, 2016), infection (Du and Arvidson, 2006), and resistance to photooxidative stress (Dufour *et al.*, 2012; Kumar *et al.*, 2012). Some species have more than one RpoH homologue, and multiplicated RpoHs appear to change promotor recognition in order to transcribe ‘heat shock’ and/or diverse functional genes (Barnett *et al.*, 2012; Dufour *et al.*, 2012; López-Leal *et al.*, 2016; Mitsui and Minamisawa, 2017). *Roseobacter denitrificans* and *D. shibae*, well-studied marine AAP species, have been shown to respond to photooxidative stress by RpoE and RpoH_II_, which is directly controlled by RpoE (Berghoff *et al.*, 2011a; Tomasch *et al.*, 2011). The results shown in Fig. 3 implied that transcripts for *rpoE* increased prior to the up-regulation of SP70 mRNA expression under carbon starvation conditions. The role of RpoE in *R. depolymerans* will be examined in the near future. SP70 appears to be the only RpoH homologue in the *R. depolymerans* genome. The function of the SP70 sigma factor appears to be related to the production of a photosystem (Fig. 4 and 5, Table 2) and heat tolerance (Table 3). However, the wild-type strain under a culture at 42°C in carbon-rich medium did not accumulate BChl *a* or carotenoid pigments (Fig. S1). Heat shock or a culture at 42°C did not trigger photosystem production, whereas carbon starvation did. Therefore, there may be other non-sigma regulatory factors (*e.g.*, factors similar to RegB-RegA, PrrB-PrrA, CrtJ, PpsR, and AppA, which respond to oxygen tension, and PpsR, AppA, AerR, and HvrA, which respond to light intensity and wavelengths) modulating the function of SP70 under heat shock and carbon starvation conditions.

Limited information is currently available on the regulatory factors for photosynthesis genes in bacteria responding to carbon starvation. The HvrA of *R. capsulatus* is an exceptional regulatory factor that may be related to the up- or downstream responses of ppGpp formation; however, the mechanisms by which HvrA controls PGC in response to nutrient stress remain unclear (Masuda and Bauer, 2004). To the best of our knowledge, SP70 of *R. depolymerans* is the first regulatory factor to be reported for the bacterial photosynthesis genes responding to the availability of carbon substrates. Soora *et al.* (2015) also showed that the RpoH_II_ of *D. shibae* was highly up-regulated in starved cells incubated in a light-dark cycle, but was not in non-starved cells, and was down-regulated in starved cells incubated in continuous dark. The RpoH homologue appears to be related to regulatory pathways responding to carbon starvation in various AAP bacteria, while its contribution to the regulation of photosynthesis genes has not yet been recognized. It remains unclear whether SP70 itself acts directly as a sigma factor to initiate the transcription of photosynthesis genes (*e.g.*, type II toxins under the control of the SP70 regulon also regulate the expression of other genes in *R. depolymerans* cells). We intend to examine the entire regulatory circuit around starvation and photosystem production in *R. depolymerans* in future studies.

## Citation

Suyama, T., Kanno, N., Matsukura, S., Chihara, K., Noda, N., and Hanada, S. (2023) Transcriptome and Deletion Mutant Analyses Revealed that an RpoH Family Sigma Factor Is Essential for Photosystem Production in *Roseateles depolymerans* under Carbon Starvation. *Microbes Environ ***38**: ME22072.

https://doi.org/10.1264/jsme2.ME22072

## Supplementary Material

Supplementary Material 1

Supplementary Material 2

## Figures and Tables

**Table 1.** Plasmids and strains used in the present study.

Strain or plasmid	Relevant characteristics	References
Strains		
61A^T^	wild-type strain of *Roseateles depolymerans*	(Suyama *et al.*, 1999)
1AA1 and 1AB1*	61A^T^ derivative, ΔSP70 by double crossover with pG19IIΔSP70K_1-A, Km^r^	This study
2CA3 and 2CA4**	61A^T^ derivative, ΔSPECF by double crossover with pG19IIΔSPECFK_2-C, Km^r^	This study
A4h	1AA1 derivative, complemented with pUC19SP70_1-2, Km^r^, Amp^r^	This study
B7h	1AB1 derivative, complemented with pUC19SP70_1-2, Km^r^, Amp^r^	This study
Plasmids		
pUC19	pMB1-type ColE1 ori, Amp^r^	(Yanisch-Perron *et al.*, 1985)
pG19II	pMB1-type ColE1 ori, pK19*mob sacB*, Gm^r^	(Maseda *et al.*, 2004)
pUC19SP70_1-2	a 1.5-kb PCR fragment containing SP70 (RD2015_RS20395) cloned in the* Kpn*I-*Xba*I site of pUC19, Amp^r^	This study
pUC19ΔSP70K_1-1	a 3.7-kb fragment from the pUC19SP70_1-2/*Msc*I-*Ngo*MIV digest ligated with a 1.3-kb kanamycin-resistant cartridge, Km^r^, Amp^r^	This study
pG19IIΔSP70K_1-A	a 2.3-kb fragment from the pUC19ΔSP70K_1-1/*Eco*RI-*Xba*I digest cloned in the *Eco*RI-*Xba*I site of pG19II, Km^r^, Gm^r^	This study
pUC19SPECF_2-7	a 1.8-kb PCR fragment containing SPECF (RD2015_RS10875) cloned in the *Kpn*I-*Xba*I site of pUC19, Amp^r^	This study
pUC19ΔSPECFK_2-6	a 3.1-kb fragment from the pUC19SPECF_2-7/*Eco*RV-*Bsp*EI digest ligated with a 1.3‍ ‍kb kanamycin-resistant cartridge, Km^r^, Amp^r^	This study
pG19IIΔSPECFK_2-C	a 1.7-kb fragment from the pUC19ΔSPECFK_2-6/*Eco*RI-*Xba*I digest cloned in the *Eco*RI-*Xba*I site of pG19II, Km^r^, Gm^r^	This study

* Strains 1AA1 and 1AB1 are two independent isolates of the ΔSP70 mutant. ** Strains 2CA3 and 2CA4 are two independent isolates of the ΔSPECF mutant.

**Table 2.** Fifty genes markedly reduced in the ΔSP70 mutant (possible member of the SP70 regulon).

Gene or deduced product	Locus	% TPM remaining in the ΔSP70 mutant
*pufA* ^§†^	RD2015_RS05160	0.71
*pufB* ^§†^	RD2015_RS05150	1.09
ORF74^§†^	RD2015_RS05155	2.34
*puhA* ^§†^	RD2015_RS04995	7.15
*hip* (HiPIP)^†^	RD2015_RS05065	9.09
*pufL* ^§†^	RD2015_RS05165	9.61
*bchY* ^§†^	RD2015_RS05140	9.62
DUF3567 domain-containing protein	RD2015_RS01775	10.58
acyl-CoA-binding protein	RD2015_RS04350	11.91
hypothetical protein	RD2015_RS20815	11.99
*bchG* ^†^	RD2015_RS05045	12.02
copper chaperone	RD2015_RS21730	12.46
*bchZ* ^§†^	RD2015_RS05145	12.46
hypothetical protein	RD2015_RS17015	12.91
hypothetical protein	RD2015_RS02360	13.39
ORF77^§†^	RD2015_RS05175	13.82
hypothetical protein	RD2015_RS03840	13.90
hypothetical protein	RD2015_RS15165	13.96
ORF440^§†^	RD2015_RS05050	14.17
RelE/ParE family toxin	RD2015_RS24285	14.24
*bchN* ^§†^	RD2015_RS05025	14.31
MFS transporter	RD2015_RS17020	14.72
hypothetical protein	RD2015_RS00640	14.82
*bchF* ^†^	RD2015_RS05030	15.02
*pufM* ^§†^	RD2015_RS05170	15.33
hypothetical protein	RD2015_RS18355	15.34
RelE/ParE family toxin	RD2015_RS00855	15.40
DUF2695 domain-containing protein	RD2015_RS17140	15.47
hypothetical protein^†^	RD2015_RS05060	15.49
iron-sulfur cluster assembly protein IscA	RD2015_RS10060	15.59
*bchC* ^§†^	RD2015_RS05130	16.09
hypothetical protein	RD2015_RS06250	16.24
hypothetical protein	RD2015_RS00990	16.26
*lhaA* ^§†^	RD2015_RS05000	16.76
NYN domain-containing protein	RD2015_RS24590	17.13
*bchX* ^§†^	RD2015_RS05135	17.72
*SP70^†^	RD2015_RS20395	17.81
*acsF* ^†^	RD2015_RS04980	18.20
*bchM* ^§†^	RD2015_RS05005	18.22
hypothetical protein	RD2015_RS05805	18.34
cysteine hydrolase	RD2015_RS03900	18.50
hypothetical protein	RD2015_RS00575	18.86
hypothetical protein	RD2015_RS16680	19.01
H-NS histone family protein	RD2015_RS07855	19.02
*bchL* ^§†^	RD2015_RS05010	19.09
hypothetical protein	RD2015_RS22185	19.11
*pufC* ^†^	RD2015_RS05180	19.21
dihydroxy-acid dehydratase	RD2015_RS13055	19.71
hypothetical protein	RD2015_RS17970	19.72
HPr family phosphocarrier protein	RD2015_RS22180	20.18

Ratio of TPMs (Wagner *et al.*, 2012) in the ΔSP70 mutant (1AA1) to those in the wild-type strain (61A). The 50 genes markedly reduced by the deletion of SP70 are listed. Loci related to PGC are shaded. ^§^ Genes also included in Fig. 2. ^†^ Genes also included in Fig. 3.

**Table 3.** Growth ability of *Roseateles depolymerans* strains in 0.4SAV medium at 30, 37, 40, and 42°C.

Genotype Strain	Wild-type		ΔSP70			SP70 complement	
	61A^T^		1AA1	1AB1		A4h	B7h
30°C	+++		+++	+++		+++	+++
37°C	++++		++	++		++++	++++
40°C	++++		–	–		++++	++++
42°C	+		–	–		+	+

++++, growth rate ≈0.55–0.69 doublings h^–1^; +++, growth rate ≈0.42–0.51 doublings h^–1^; ++, growth rate ≈0.12–0.13 doublings h^–1^; +, growth rate ≈0.09–0.11 doublings h^–1^; –, no growth observed.
