# Supplementary material for: Transcriptome and Deletion Mutant Analyses Revealed that an RpoH Family Sigma Factor Is Essential for Photosystem Production in Roseateles depolymerans under Carbon Starvation
Source: Microbes Environ. 2023 Mar 7;38(1):ME22072. doi: 10.1264/jsme2.ME22072 (PMC10037100; doi:10.1264/jsme2.ME22072)
Supplement: Supplementary file 1 — Supplementary Material 1 [file 38_22072_s1.pdf]

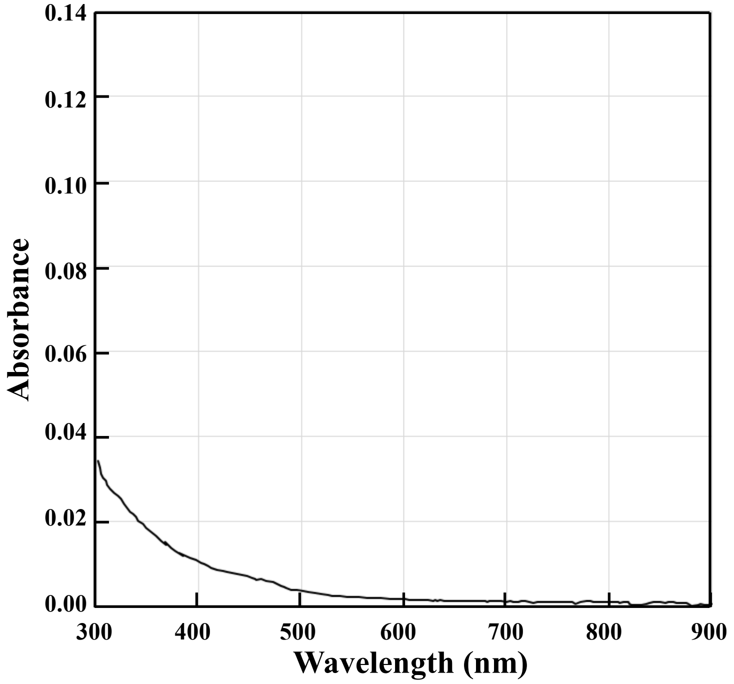

Fig. S1. An absorption spectrum of a methanol extract from cells of the wild-type strain, 61A^T at stationary phase grown in 0.4SAV at 42˚C in the dark for 70 h. The intensity of spectrum was normalized for the dry weight of the sample cells so that this can be compared with Figs. 5A–C.
